# Supplementary material for: PFN2 and NAA80 cooperate to efficiently acetylate the N-terminus of actin
Source: J Biol Chem. 2021 Jan 13;295(49):16713–31. doi: 10.1074/jbc.RA120.015468 (PMC7864067; doi:10.1074/jbc.RA120.015468)
Supplement: Supplementary file 1 [file mmc1.zip › 162624_2_supp_601324_qh2w34.pdf]

Supporting Information

Ree et al. - ***PFN2 and NAA80 cooperate to efficiently acetylate the N-terminus of actin***

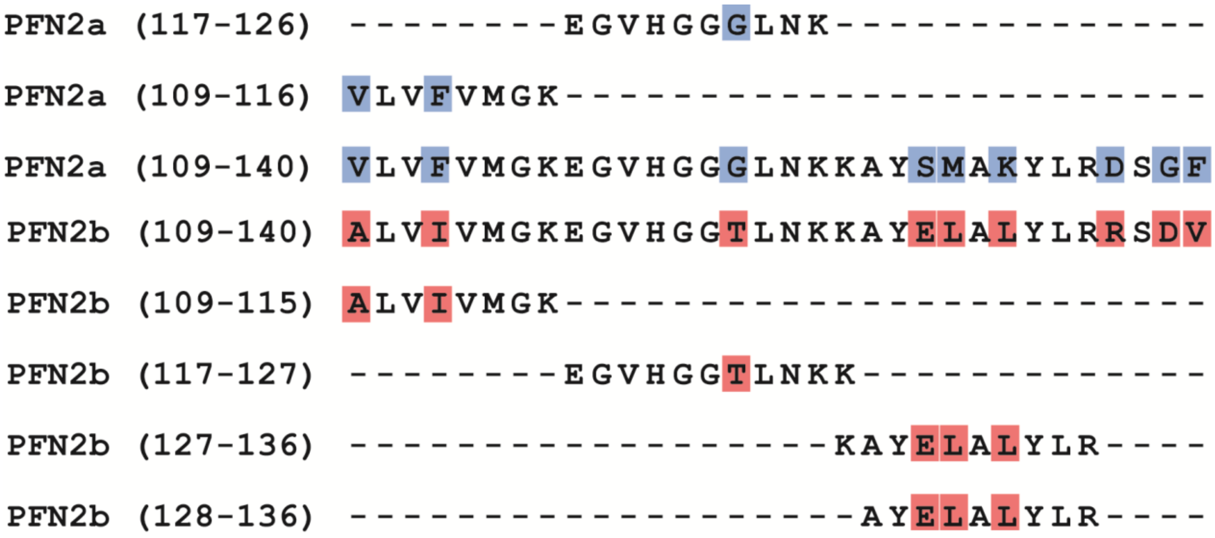

Figure S1: PFN2 isoform specific peptides identified in at least one NAA80-V5 IP aligned with the variable region of PFN2a and PFN2b (aa 109-140). The differing sites are labeled with blue (PFN2a) or red (PFN2b).

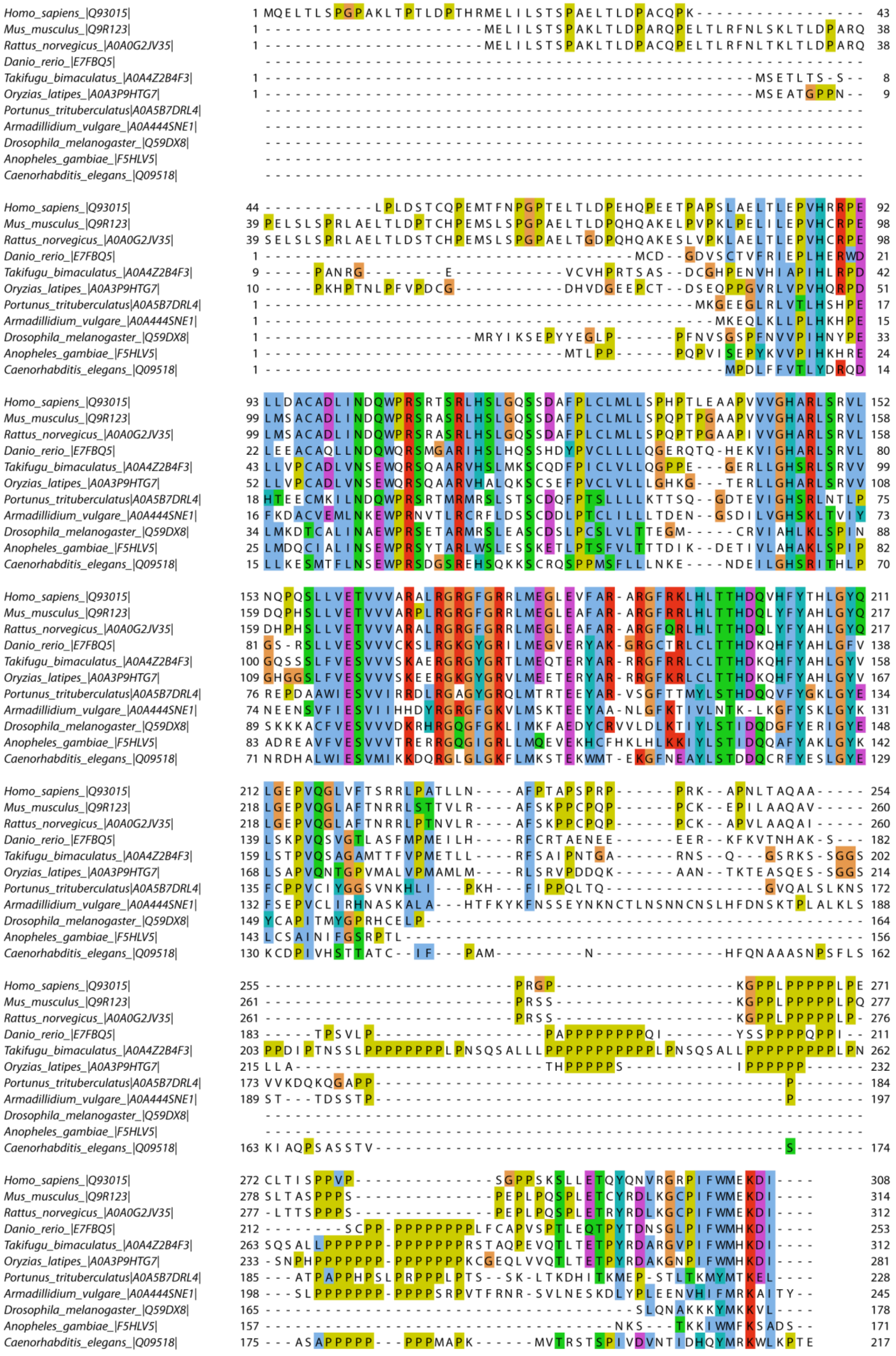
Homo_sapiens_|Q93015|
Mus_musculus_|Q9R123|
Rattus_norvegicus_|A0A0G2JV35|
Danio_rerio_|E7FBQ5|
Takifugu_bimaculatus_|A0A4Z2B4F3|
Oryzias_latipes_|A0A3P9HTG7|
Portunus_trituberculatus|A0A5B7DRL4|
Armadillidium_vulgare_|A0A444SNE1|
Drosophila_melanogaster_|Q59DX8|
Anopheles_gambiae_|F5HLV5|
Caenorhabditis_elegans_|Q09518|

Figure S2: NAA80 alignment. NAA80 protein sequences from *Homo sapiens* (Q93015), *Mus musculus* (Q9R123), *Rattus norvegicus* (A0A0G2JV35), *Danio rerio* (E7FBQ5), *Takifugu bimaculatus* (A0A4Z2B4F3), *Oryzias latipes* (A0A3P9HTG7), *Portunus trituberculatus* (A0A5B7DRL4), *Armadillidium vulgare* (A0A444SNE1), *Drosophila melanogaster* (Q59DX8), *Anopheles gambiae* (F5HLV5) and *Caenorhabditis elegans* (Q09518). Sequences were retrieved from UniProt (identifiers in parentheses) and aligned with Clustal Omega.

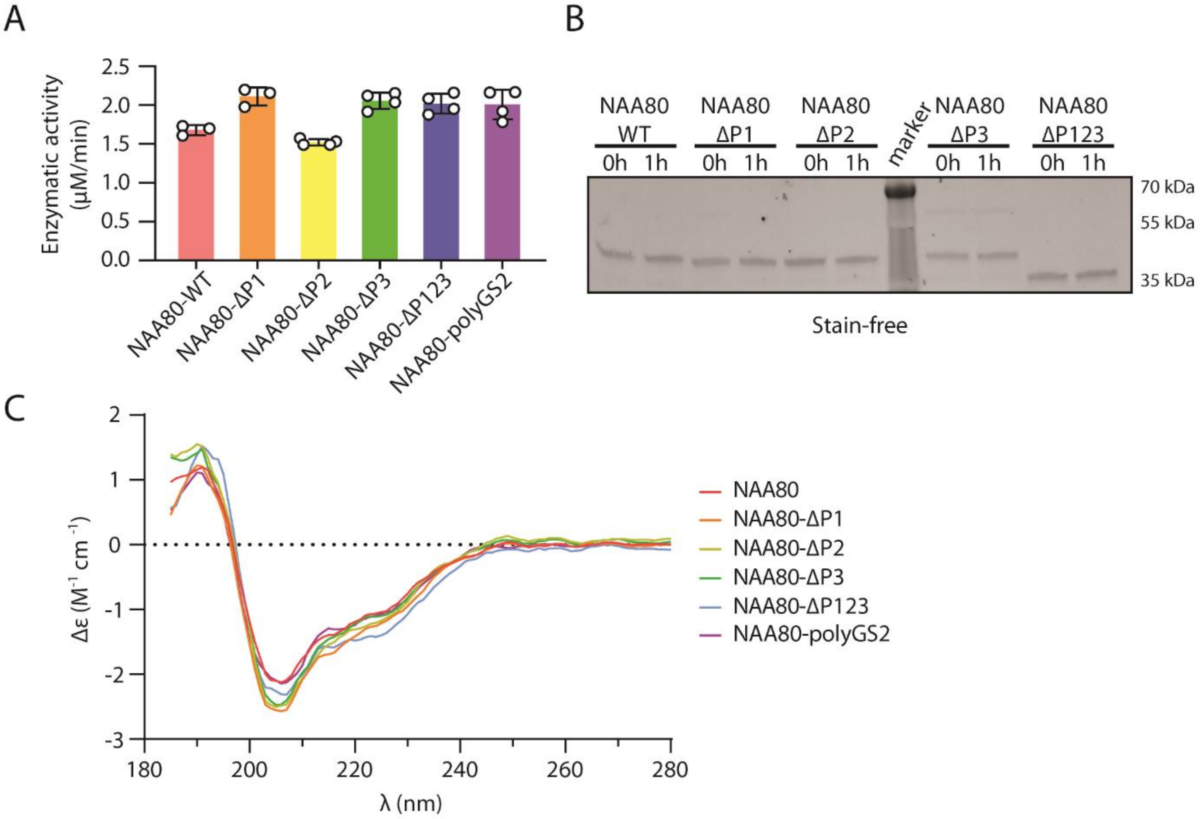

Figure S3: Basal activity and stability of NAA80-WT and polyproline mutants. A) Enzymatic activity of the indicated NAA80 variants were measured using the DTNB assay (n = 3 for NAA80-WT and NAA80-ΔP1, n = 4 for other variants) for all variants). B) Enzymes used in S3a were resolved on an SDS-PAGE gel. C) CD spectra for NAA80 variants.

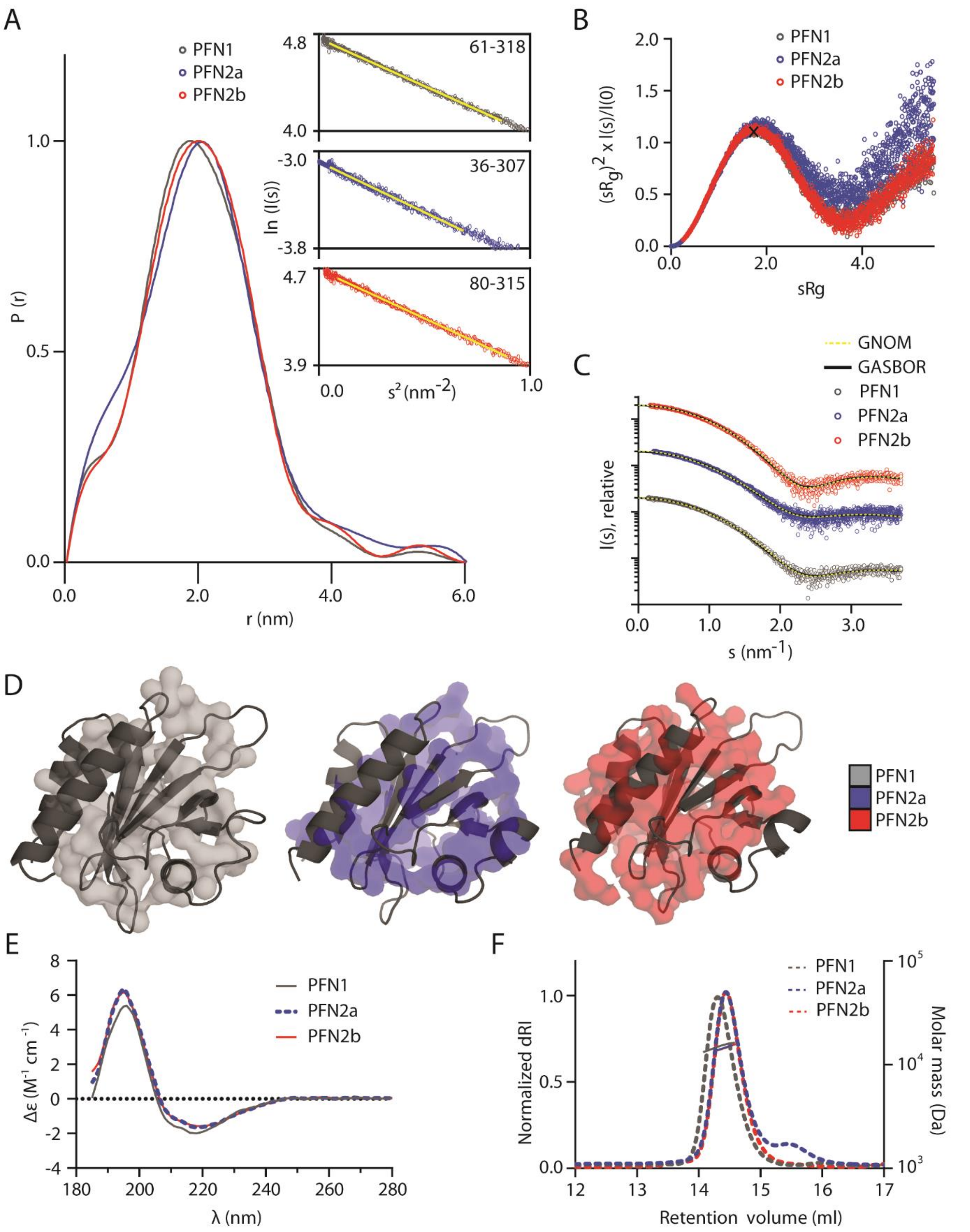

Figure S4: SAXS, CD and MALS measurements of profilins. A) Distance distribution of SAXS measurements of PFN1, PFN2a and PFN2b. Insert: Guinier plots of the same proteins. B) Dimensionless Kratky plot for PFN1, PFN2a, and PFN2b. The cross indicates the expected maximum for a rigid, spherical particle. C) Scattering profiles of PFN1, PFN2a and PFN2b. The data were shifted along the y-axis for clarity. The yellow, dotted line shows the GNOM fit, while the black line represents the GASBOR fit. D) GASBOR models of PFN1 (grey), PFN2a (blue) and PFN2b (red) overlaid with the crystal structures of each protein (surface, PFN1 PDB: 1FIK, PFN2 PDB: 1D1J). E) CD data for PFN1, PFN2a and PFN2b. F) SEC-

MALS analysis of PFN1, PFN2a and PFN2b. Dotted lines show the differential refractive index recorded over the course of the SEC run (12 - 17 ml), while solid lines show the molecular mass of the particles in the peak.

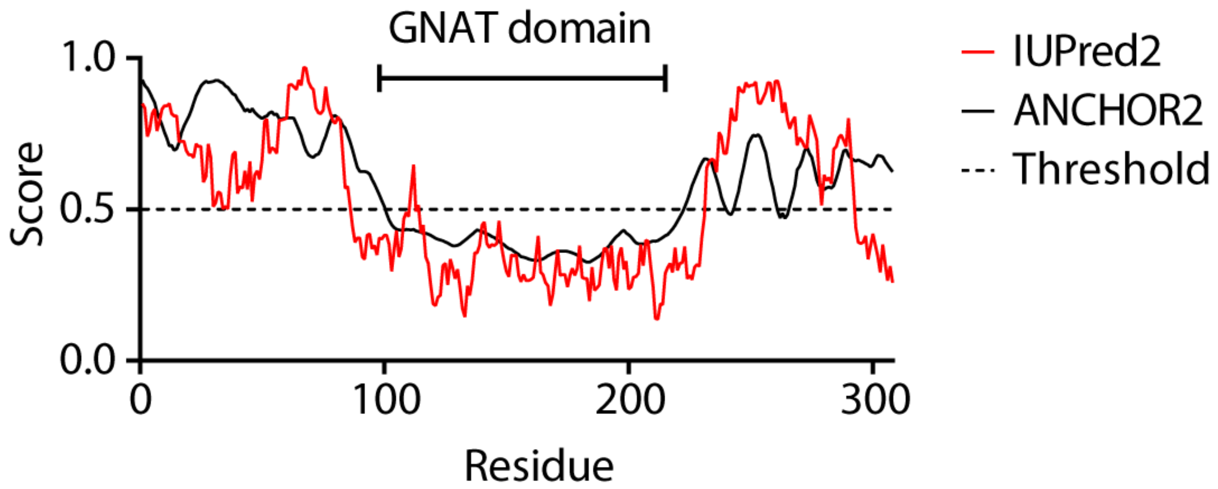

Figure S5: Protein disorder prediction for NAA80, generated by IUPred2 and ANCHOR2 predictors.

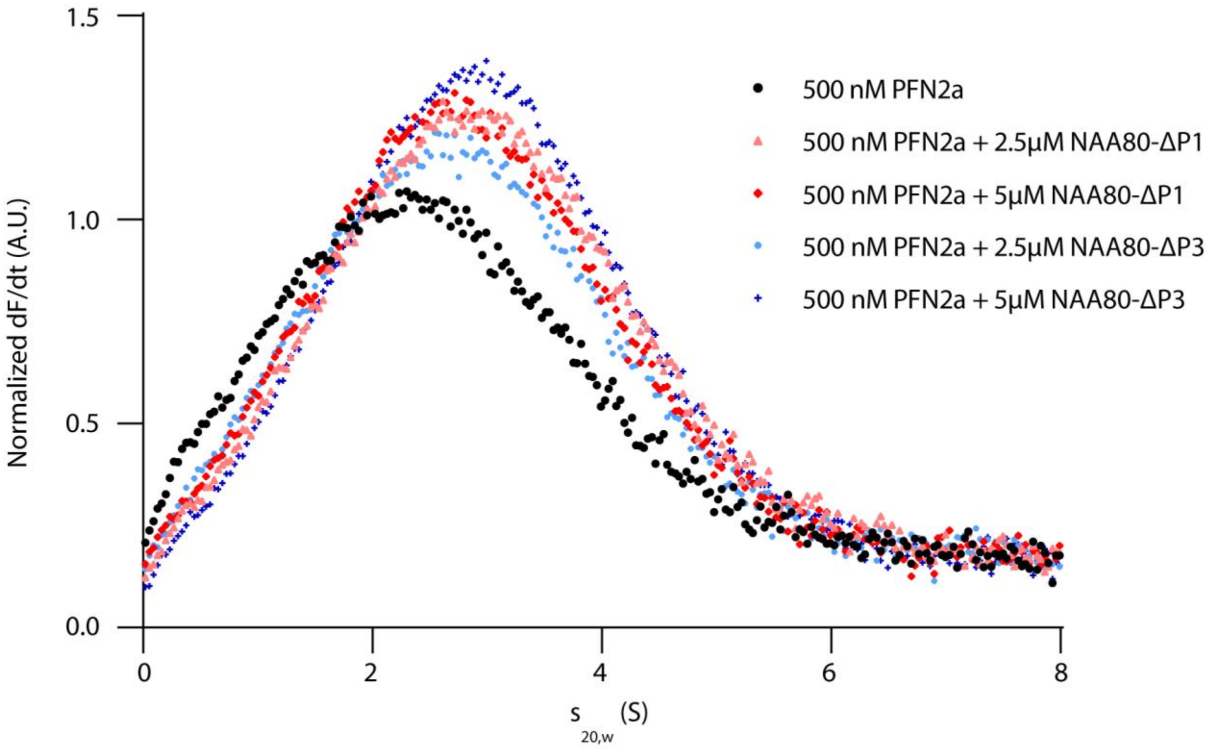

Figure S6: Titration of NAA80-ΔP1 and NAA80-ΔP3 to PFN2a in AUC experiments. AUC with labeled PFN2a and NAA80 deletion mutants, NAA80-ΔP1 or NAA80-ΔP3, in different concentrations.

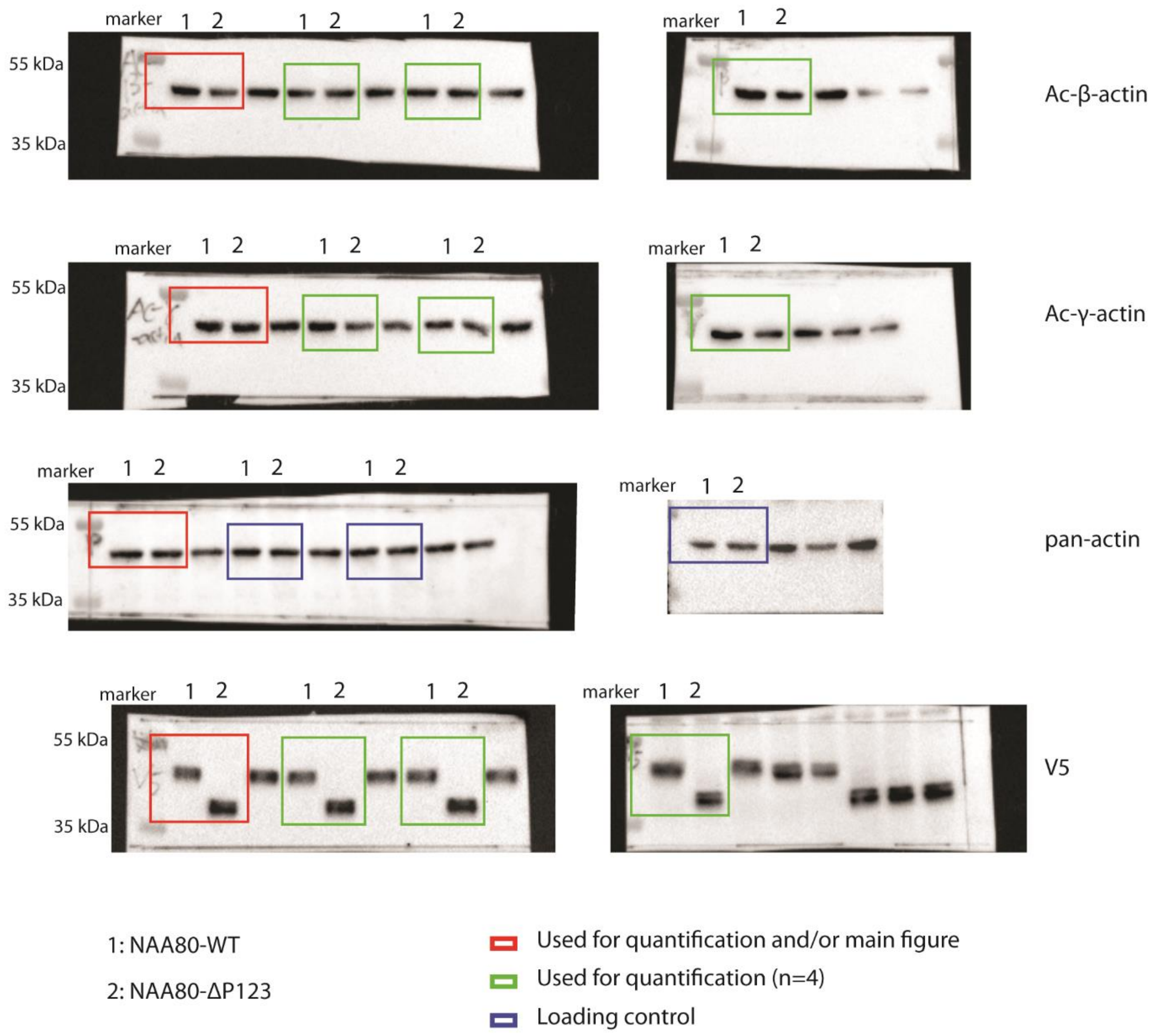

Figure S7: Supplemental blots for the *in vivo* NAA80-WT and NAA80-ΔP123 actin reacetylation assay (Fig. 5C). *NAA80*-KO cells were transfected with NAA80-WT or NAA80-ΔP123 (n = 4) for 14 hours and the lysates were probed with the indicated antibodies. Blots used in quantification (Ac-β-actin, Ac-γ-actin and V5) or as loading control (pan-actin) and included in the main figure (Fig. 5C) are outlined in red. Blots used only for quantification are outlined in green, and loading controls are outlined in blue.

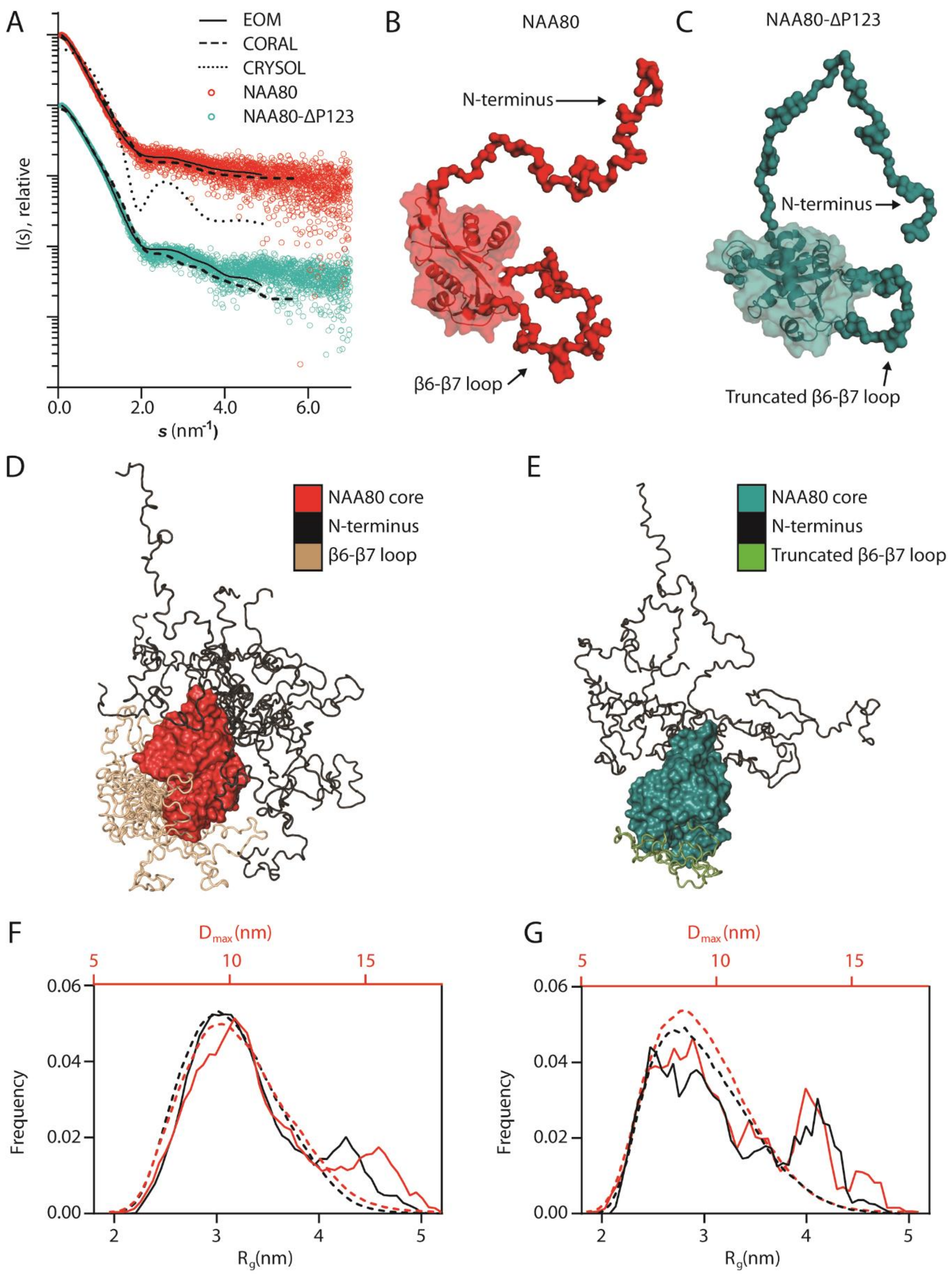

Figure S8: Structural information on full-length NAA80 and NAA80-ΔP123 obtained from SAXS. A) Scattering profiles of NAA80 and NAA80-ΔP123. The scattering data (dots) have been offset along the y-axis for clarity. Data fits are shown as black lines. Fit and data legend is shown as inset. The structure used for comparison by CRYSOL was *Dm*NAA80 (PDB: 5WJD(36)). Rigid body SAXS models with missing residues modeled as dummy atoms were generated for NAA80 (B) and NAA80-ΔP123 (C) by using CORAL. D-E) EOM conformers of NAA80 (D) and NAA80-ΔP123 (E).

F-G) D_{max} (red) and R_g (black) distributions from EOM analysis with ensemble frequencies (solid line) and pool frequencies (dashed lines) for NAA80 (F) and NAA80-ΔP123 (G).

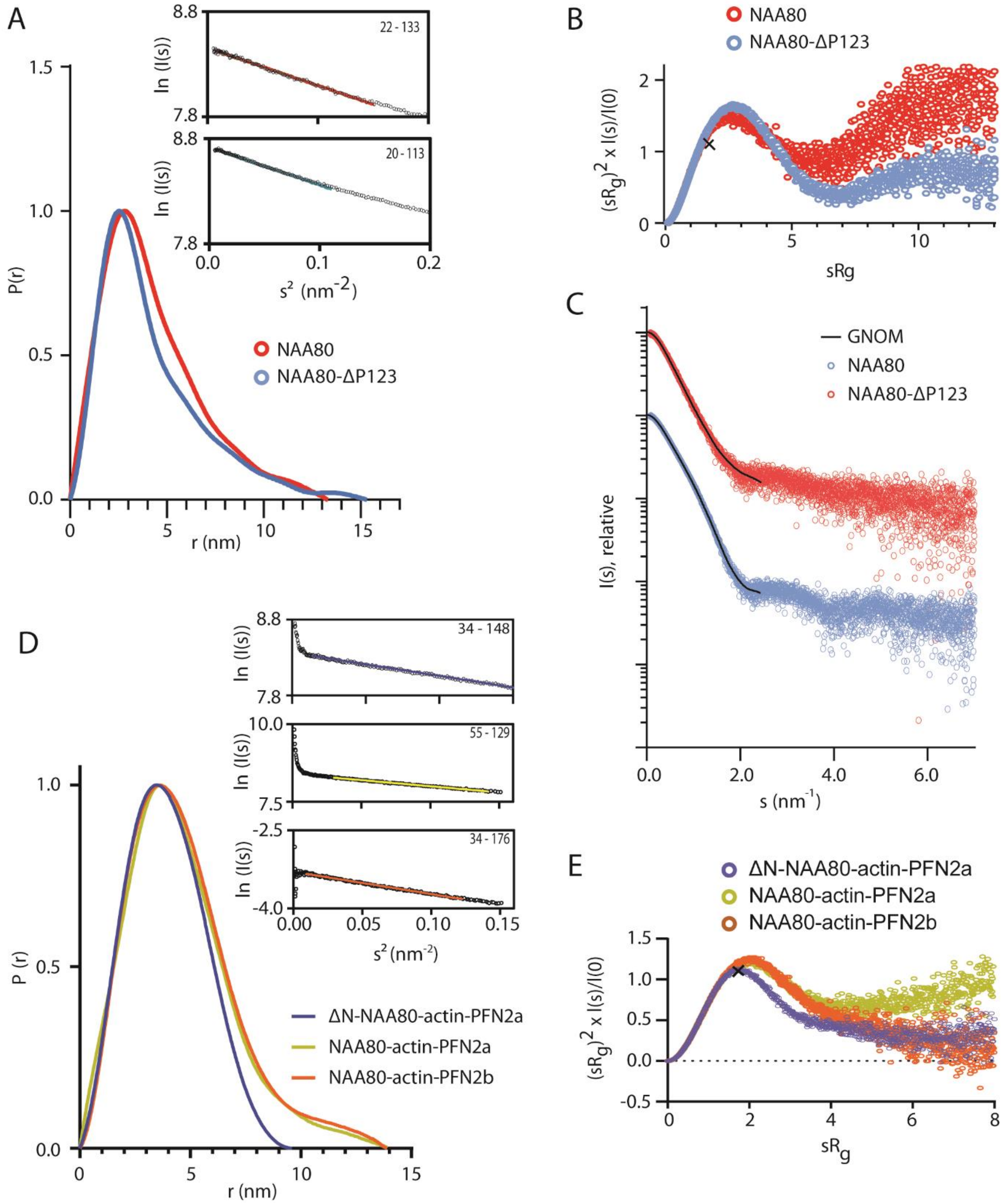

Figure S9: SAXS measurements of NAA80, NAA80-ΔP123, ΔN-NAA80-actin-PFN2a, and NAA80-actin-PFN2. A) Distance distribution of SAXS measurements of NAA80 and NAA80-ΔP123. Inset: Guinier plots of the same proteins. B) Dimensionless Kratky plots for NAA80 (red) and NAA80-ΔP123 (blue). The cross indicates the expected maximum for a rigid, spherical particle. C) Scattering profiles of NAA80 (red) and NAA80-ΔP123 (blue). The data were shifted along the y-axis for clarity. The black line represents the GNOM fit (solid line). D) Distance distribution functions for ΔN-NAA80-actin-PFN2a (blue), NAA80-actin-PFN2a (yellow), and NAA80-actin-PFN2b (orange), with Guinier plots shown as inset. E) Normalized Kratky analysis plots for the same complexes. The cross indicates the expected maximum for a rigid, spherical particle.

Tables S1 and S2: Excel sheets contain quantified proteins from LC/MS interactor screens in HeLa cells (Table S1) and HAP1 cells (Table S2). Available for download online with this article. The mass spectrometry proteomics data and Tables S1-S2 have additionally been deposited to the ProteomeXchange Consortium via the PRIDE partner repository with the dataset identifier PXD021408.

Table S3: Thermal stability of NAA80 variants determined by DSF or CD (*)

**Protein**	**T_m (°C)**	***s* (°C)**
NAA80	41.75	0.07
NAA80-ΔP1	42.65	0.25
NAA80-ΔP2	42.15	0.25
NAA80-ΔP3	41.28	0.07
NAA80-ΔP123	41.06	0.17
NAA80-PolyGS2*	41.00	0.45

Table S4: SAXS parameters for PFN1, PFN2a, and PFN2b

**Data collection**			
	**PFN1**	**PFN2a**	**PFN2b**
Instrument	P12, PETRA III, DESY, Hamburg	B21, Diamond Light Source, Harwell	P12, PETRA III, DESY, Hamburg
Wavelength (nm)	0.124	0.100	0.124
Angular range (nm^{-1})	0.04 – 7.3	0.04 – 3.7	0.04 – 7.3
Temperature (°C)	10	10	10
SAXS mode	Batch	Batch	Batch
Exposure time (s)	0.045	1	0.045
Concentration (mg/ml)	1.3 – 5.2	1.1 – 4.5	1.4 – 5.7
**Software**			
Primary data reduction and processing	PRIMUS		
Data validation and analysis	PRIMUS		
Ab initio modelling	GASBOR		
Graphical representation	PyMOL		
**Structural data**			
I(0) (relative), from Guinier	107.2	0.05	107.2
R_g (nm), from Guinier	1.60	1.61	1.59
s-range (nm^{-1}) used in Guinier	0.22 – 0.93	0.23 – 0.83	0.30 – 0.95
I(0) (relative), from P(r)	107.2	0.05	107.2
R_g (nm), from P(r)	1.60	1.61	1.60
D_{max} (nm), from P(r)	6.0	6.0	6.0
s-range (nm^{-1}) used in P(r)	0.15 – 5.19	0.20 – 5.10	0.33 – 3.7
**Molecular weight determination**			
M_r (kDa), theoretical from sequence	15.05	15.05	15.09
M_r (kDa), from I(0)	14.30	14.60	14.30
***Ab initio* modelling**			
χ^2	1.76	1.26	1.68

Table S5: SAXS parameters for NAA80 and ternary complexes

**Data collection parameters**					
	**NAA80**	**NAA80-ΔP123**	**ΔN-NAA80-actin-PFN2a**	**NAA80-actin-PFN2a**	**NAA80-actin-PFN2b**
Instrument	P12, PETRA III, DESY, Hamburg			B21, Diamond Light Source, Harwell	
Wavelength (nm)	0.124			0.1	
Angular range (nm^{-1})	0.02 – 7.32			0.03 – 4.4	
SAXS mode	SEC-SAXS			SEC-SAXS	
Injection volume (µl)	100			45	
Exposure time (s/frame)	1			2.3	
Number of frames	3000			1259	
Temperature (°C)	10			10	
**Software**					
Data analysis software	ATSAS 2.8				
Primary data reduction	CHROMIXS, PRIMUS				
Rigid body modelling	CORAL				
Ensemble optimization modelling	EOM				
Generation and fitting of theoretical scattering profiles	CRYSOL				
Ab initio modelling	GASBOR				
Graphical representation	PyMOL				
**Structural parameters**					
I(0) (relative), from Guinier	4695	6115	4273	4425	0.037
R_g (nm), from Guinier	3.27	3.25	3.04	3.44	3.58
I(0) (relative), from P(r)	4747	6181	4273	4485	0.04
R_g (nm), from P(r)	3.47	3.49	3.04	3.59	3.70
D_{max} (nm), from P(r)	13.3	15.3	9.58	13.90	13.85
Quality estimate	0.71	0.66	0.98	0.79	0.75
**Rigid body modelling**					
Crystal structure	6NAS:B	6NAS:B		6NAS	6NAS
Flexible residues in NAA80	1 – 79, 217 – 303	1 – 79, 217 – 255 (256 – 302 missing)		1 – 80, 221 – 258, 273 – 289	1 – 80, 221 – 258, 273 – 289
χ^2	3.25	23.1		1.45	1.16
**Ensemble optimization method**					
Crystal structure	6NAS:B	6NAS:B			
Flexible residues in NAA80	1 – 79, 217 – 303	1 – 79, 217 – 255 (256 – 302 missing)			
Number of conformers	10	6			
R_g (nm)	3.35	3.20			
D_{max} (nm)	11.16	10.46			
χ^2	0.95	1.45			
**Generation of theoretical scattering profile and SAXS profile fitting**					

Crystal structure	5WJD		6NAS		
R_g (nm)	1.65		3.06		
D_{max} (nm)	5.2		9.88		
χ^2	72.16		1.28		
***Ab initio* modelling**					
χ^2			1.63		

Table S6: List of primers, plasmids, antibodies and peptides used in this study.

Primer ID	Sequence	Plasmid product/Usage
oTA22: NAA80 KozF	caacatgcaagagctgactc	pTA166
oTA792: NAA80 w STOP rv	tcagatgtctttttccatccagaatatg	pTA166
oTA732: NAA80 fw	Atgcaagagctgactctgagc	pTA538
oTA311: NAA80 w STOP	Catgaattccattcagatgtctttttccatcc	pTA538
oTA812: NAA80 M23L fw	tacacacccggctggagctgat	pTA587, introducing M23L
oTA813: NAA80 M23L rv	Gggtctagtgtaggggtc	pTA587, introducing M23L
oTA864: pcDNA4 Xpress to V5 fw	ctcctcggtctcgattctacggtacaggccccttatgcaag	pTA609, replacing N-terminal Xpress tag with V5 tag
oTA865: pcDNA4 Xpress to V5 rv	agggttagggataggcttaccccgacccatttgctgtcc	pTA609, replacing N-terminal Xpress tag with V5 tag
oTA904: NAA80□238-247 fw	aacctgactgcccaagctg	pTA646, pTA777, pTA780
oTA905: NAA80□238-247 rv	Ggctgtggggaaggcatt	pTA645, pTA777
oTA906: NAA80□277-284 fw	Tcaaaaagcctgctggag	pTA779
oTA907: NAA80□277-284 rv	Tgagatggtcaggcactc	pTA779, pTA780
oTA917: NAA80□260-270 fw	gagtgcctgaccatctcaa	pTA778
oTA918: NAA80□260-270 rv	cttgggaccccttggggca	pTA778
oTA1146: NAA80 polyGS2 fw	ggctctggcctatctgagtgcctgaccatctcac	pTA858
**oTA1147: NAA80 polyGS2 rv**	**agagcccaagctgcctcccttgggaccccttgg**	**pTA858**

-

Plasmid ID	Description	Expression	Protein
pTA12	pcDNA3.1-lacZ-V5	Mammalian	β-galactosidase-V5
pTA166	pcDNA3.1-NAA80-V5	Mammalian	NAA80-V5
pTA538	pcDNA4-Xpress-NAA80	Mammalian	Xpress-NAA80
pTA587	pcDNA4-Xpress-NAA80-M23L	Mammalian	Xpress-NAA80 (M23L)
pTA598	pTYB11-profilin-1	Bacterial	PFN1
pTA599	pTYB11-profilin-2a	Bacterial	PFN2a
pTA600	pTYB11-profilin-2b	Bacterial	PFN2b
pTA609	pcDNA4-V5-NAA80	Mammalian	V5-NAA80 (M23L)
pTA646	pcDNA4-V5-NAA80-Δ238-284	Mammalian	V5-NAA80 (M23L) Δ238-284 (ΔP123)
pTA631	pCold1-Gelsolin C'term half (G4-G6)	Bacterial	Gelsolin
pTA858	pcDNA4-V5-NAA80-polyGS2	Mammalian	V5-NAA80 (M23L) polyGS2
pTA751	pTYB12-NAA80	Bacterial	NAA80
pTA777	pTYB12-NAA80-Δ238-247	Bacterial	NAA80-Δ238-247 (ΔP1)
pTA778	pTYB12-NAA80-Δ260-271	Bacterial	NAA80-Δ260-271 (ΔP2)
pTA779	pTYB12-NAA80-Δ277-284	Bacterial	NAA80-Δ277-284 (ΔP3)
pTA750	pTYB12-ΔN-NAA80	Bacterial	ΔN-NAA80

PRIMARY ANTIBODIES				
**Antibody**	**Cat. Nr**	**Company**	**Animal**	**Dilution**
Pan-actin	AAN01	Cytoskeleton	Rabbit	1:2000
Pan-actin	Ab14128	Abcam	Mouse	1:2000
Ac-β-actin	ab6276	Abcam	Mouse	1:3000
Ac-γ-actin	ab123034	Abcam	Mouse	1:5000
Profilin-1 (PFN1)	ab50667	Abcam	Rabbit	1:2000
Profilin-2 (PFN2)	SC-100955	Santa Cruz	Mouse	1:1000 - 1:2000
V5	R960CUS	Invitrogen	Mouse	1:1000 - 1:5000
α-Vinculin	ab129002	Abcam	Rabbit	1:1000 - 1:2000
NAA80	custom	Biogenes	Rabbit	1:200

SECONDARY ANTIBODIES AND STAINING REAGENTS					
**Antigen**	**Cat. Nr**	**Company**	**Animal**	**Dilution**	**Usage**
ECL Anti-mouse IgG-HRP	NA931	Amersham	Goat	1:3000-1:20000	Western blotting
ECL Anti-rabbit IgG-HRP	NA934	Amersham	Goat	1:3000-1:20000	Western blotting

Name	Oligopeptide sequence	NAT	Derived from	UniProt
DDDI	[H] **DDDIAAL** RWGRPVGRRRRPVRVYP [OH]	H	β-cyto-actin	P60709
EEEI	[H] **EEEIAAL** RWGRPVGRRRRPVRVYP [OH]	H	γ-cyto-actin	P63261
